# Immunity in the ABM-DSGE Framework for Preventing and Controlling Epidemics—Validation of Results

**DOI:** 10.3390/e24010126

**Published:** 2022-01-14

**Authors:** Jagoda Kaszowska-Mojsa, Przemysław Włodarczyk, Agata Szymańska

**Affiliations:** 1 Institute for New Economic Thinking at the Oxford Martin School, University of Oxford, Manor Road, Oxford OX1 3UQ, UK; 2 Institute of Economics, Polish Academy of Sciences, Nowy Świat St. 72, 00-330 Warsaw, Poland; 3 Department of Macroeconomics, Institute of Economics, Cracow University of Economics, Rakowicka St. 27, 31-510 Cracow, Poland; 4 Department of Macroeconomics, Faculty of Economics and Sociology, University of Łódź, 90-136 Łódź, Poland; przemyslaw.wlodarczyk@uni.lodz.pl (P.W.); agata.szymanska@uni.lodz.pl (A.S.)

**Keywords:** COVID-19, vaccination, agent-based modelling, dynamic stochastic general equilibrium models, scenario analyses, validation of results

## Abstract

The COVID-19 pandemic has raised many questions on how to manage an epidemiological and economic crisis around the world. Since the beginning of the COVID-19 pandemic, scientists and policy makers have been asking how effective lockdowns are in preventing and controlling the spread of the virus. In the absence of vaccines, the regulators lacked any plausible alternatives. Nevertheless, after the introduction of vaccinations, to what extent the conclusions of these analyses are still valid should be considered. In this paper, we present a study on the effect of vaccinations within the dynamic stochastic general equilibrium model with an agent-based epidemic component. Thus, we validated the results regarding the need to use lockdowns as an efficient tool for preventing and controlling epidemics that were obtained in November 2020.

## 1. Introduction

Last year was dominated by discussions on how to contain the spread of the Sars-CoV-2 virus and the economic impact of the prevention and control measures. The need to effectively introduce lockdowns has been discussed in the literature, in particular, when there had not yet been widespread vaccinations and there was no consensus on how to treat patients who had contracted COVID-19. It was also unclear how contagious the virus was and whether it was possible to get re-infected or whether the body developed an immunity against the virus. While many open questions have already been answered since the outbreak of the pandemic, some crucial questions are still unanswered. It is especially worth considering how justified and effective lockdowns are in the era of widespread vaccinations against COVID-19.

In our article [1], which was published in November 2020, we studied the shape and range of state interventions whose goal was to limit the negative effects of the pandemic, in particular, by reducing the number of infections and deaths that were caused by the pandemic. As it was emphasised, lockdowns have been introduced in many countries around the world in order to limit the spread of the virus and to prevent the collapse of the health systems. In some countries, a deep lockdown strategy was abruptly adopted, while in others, the focus was put on gradually closing certain sectors of the economy. The effectiveness of a lockdown in a given country is influenced by many factors, the most important of which are legal, behavioural, cultural and social factors. Hence, the impact of a lockdown on the course of the epidemic and its impact on the given economy could be different than in other countries. In our previous paper, we advised on how the lockdown policy should be implemented. In this article, we would like to validate the results taking into account that the COVID-19 vaccinations started in most developed countries in 2021. Therefore, we address the same two major questions:

- Should we freeze an economy in order to decrease the pace of SARS-CoV-2 transmission?
- What should the scale and composition of an efficient lockdown policy look like?

However, this time, we did take immunity into account in our analysis.

## 2. Literature Review

The efficiency of the COVID-19 vaccination process in the context of the potential to achieve herd immunity by a society is one of the most frequently discussed topics in the literature today. In simple terms, “herd immunity works through achieving a threshold immunity at the population level that is able to theoretically cut the transmission chain of a given infectious disease, be it obtained through natural infection or vaccination” [2]; for general studies on the topic, see also [3,4,5,6]. In the most general terms, two main sources of achieving herd immunity were discussed—through a natural infection and recovery or by a vaccination [3].

In the literature, the results of many studies whose goal was to compute the thresholds for infectious diseases have been presented, including recent works that have focused on the threshold for the COVID-19 disease. Fontanet and Cauchemez [6] suggested that under the assumption about the absence of control measures $\left(p_{c}=0\right)$, i.e., without pharmacological interventions, among others, “the condition for herd immunity (R<1, where $R=\left(1-p_{i}\right)R_{o})$ is attained when the proportion of immune individuals reaches $p_{i}=1-\frac{1}{R_{o}}$, where $R_{o}$ denoted the reproduction number in the absence of control measures in a fully susceptible population” and that it can vary across populations and over time. In the formula above, which was presented by [6], *R* denotes the effective reproduction number that is explained as “the average number of persons infected by a case”. As is clarified, in the absence of interventions, the number *R* is lower than 1 and this case denotes the possibility of the occurrence of herd immunity, i.e., a situation in which one infected person is responsible for inducing “less than one secondary case on average”, see [6]. The rest of the abbreviations that are used by the authors in the formula explaining herd immunity are as follows: $p_{i}$ is the proportion of the society that is immune and $p_{c}$ denotes the relative reduction in the transmission rates that is achieved by using non-pharmaceutical interventions.

The estimates for the COVID-19 pandemic differ. As was mentioned in [6], $R_{o}$ varies across populations and over time. The literature review emphasises the differences across countries and regions. For example, Kwok et al. [7], in a sample of 32 countries, estimated that the effective reproduction number ranged from 1.06 in Kuwait to 6.64 in Bahrain as of 13 March 2020. As a result, the minimum proportion of the total population, in percentage terms, that would be required to recover from COVID-19 was between 5.66% in Kuwait to 85% in Bahrain, see [8]. As was emphasised by Fontanet and Cauchemez [6], in the case of SARS-CoV-2, most estimates of $R_{o}$ are in the range of 2.5 and 4. Moreover, Kwok et al. [7] obtained the $R_{o}$ between 2 and 4 for the largest group of countries. For $R_{o}=2$, which they estimated for Iran, the herd immunity threshold for SARS-CoV-2 was expected to require 50% of the population to have immunity, for $R_{o}=2.09$, while the threshold increased to 65.5% for the UK. The estimates for Israel (a country with one of the widest distributions of vaccines against COVID-19 among its population, [9]), the $R_{o}$ was 3.02 and the estimated threshold was 66.9%. As has been argued, the low figures for Kuwait reflected the fact that the country had strong lockdowns and put many measures in place to control the SARS-CoV-2 virus and an escalation of the COVID-19 pandemic [10]. Aschwanden in her article [10] emphasised that the threshold for herd immunity, taking into account the literature overview, ranged from 10% to more than 70%. However, in the most compelling research, it has ranged between 60 and 70% [10]. Gomes et al. [11] indicated that the initial and simple estimates of a COVID-19 threshold that were based on relying on homogeneity assumptions ranged between 60 and 80% of a population to become immune. In their study, which was based on the earlier models that had been explored in the literature, but with the individual variations in susceptibility or in exposure to infection incorporated into the data, they obtained a lower herd immunity threshold, for example, for SARS-CoV-2, they calculated the threshold that was associated with a natural infection to be in the range of 10–30%. As has been presented, in order to obtain the required threshold for herd immunity, the specified size of the proportion of the population to have immunity is required.

The thresholds of herd immunity that have been presented in the literature are influenced by the vaccination process and vaccine coverage. Using the SMEIHRDV model, Dashtbali and Mirzaie [12] predicted that the number of infected cases at the height of the COVID-19 pandemic was significantly reduced by the increasing vaccine coverage of between 0.2 and 0.6. Their results were predicted for Egypt and Germany. In the case of Egypt, the number of infected cases at the height of the epidemic was estimated to decrease from around 540 cases with a vaccine coverage of 0.2 to around 200 cases when the vaccine coverage increased to 0.6. In the case of Germany, the obtained predictions suggested that increasing the vaccine coverage from 0.2 to 0.6 affected the pandemic peak, in which the cases decreased from approximately 320 to 120. Moreover, the German case enabled it to be predicted that, both investing in a strategy of social distancing and increasing the vaccine coverage, the length of the epidemic peak of infected cases might shorten from approximately 200 to 55 by increasing vaccine coverage from 0.2 to 0.6.

The results obtained by Makhoul et al. [13] indicated that even a partially efficient vaccine was able to affect the spread of SARS-CoV-2 virus and the COVID-19 pandemic. The authors calibrated and estimated their model for the Chinese case and assumed a long duration of vaccine protection that lasted ten years. As was argued, the simulated scenarios emphasised that the three vaccines do not need to provide complete immunity to be able to completely control the infection. As it was also emphasised, a vaccine with $VE_{s}\geq 70\%$ (i.e., a vaccine efficacy in reducing susceptibility of greater than 70%) would have enabled us to control the pandemic at ≥80% coverage before its onset. However, as was estimated, when the reproduction number $R_{o}$ is assumed to be three, then the minimum $VE_{s}$ that is required to eliminate infections is about 90%.

The simulations of Charumilind et al. [14] for the US and the UK showed that the use of a vaccine that is 95% effective at preventing transmission and with a natural immunity between 5–20%, the required vaccine coverage had to increase when more than a 40–80% transmissible COVID-19 strain was predominant, and more stringent non-pharmaceutical interventions should be used to manage the pandemic. For example, under the assumption that a COVID-19 strain is 40% more transmissible, then the required coverage for the two countries would be 65–72% (or 78–86% if limited only to those more than 12 years old). However, when a new COVID-19 strain was assumed to be 80% more transmissible, then the required coverage should be increased and it would be 75–80% (or 89–95% if limited to only those more than 12 years old). As was investigated, if the transmissibility of a new variant increased by 40% or 80%, then COVID-19 herd immunity can only be achieved once the total immune population reaches 70% (under assumption $R_{o}=3.4$) or 77% (with $R_{o}=4.3$).

Using a deterministic model, Han et al. [15] analysed “the connection between the daily vaccination capacity (rollout speed) and transmissibility in determining the optimal (vaccine prioritisation) strategies” based on the case of China. They argued that introducing a high vaccination capacity in the early phase of a vaccination campaign is crucial for achieving large increases in strategic prioritisations. The simulations were based on a time-varying optimisation of the COVID-19 vaccine prioritisation. The obtained findings enabled them to conclude that increasing the vaccination capacity to 2.5 million first doses per day (0.17% roll-out speed) or higher could considerably reduce the COVID-19 burden, when the assumed reproduction number was equal to 1.5 $\left(R_{o}=1.5\right)$.

The Chen et al. [16] study was based on eight selected countries: Chile, Hungary, Israel, Serbia, Qatar, the UAE, the UK and the USA. These countries were selected due to their high degree of effectiveness in mitigating the COVID-19 pandemic in a situation in which the rate of vaccination achieved the level of criticality, even if it was lower than the herd immunity threshold. The results of the research suggested that a value of the vaccination rate of 50.91 doses per 100 people could be perceived as the minimum condition for avoiding an exacerbation of the pandemic in a society.

Coccia’s [8] study, which was based on 192 countries with data from March to May 2021, indicated that the optimal levels of vaccination in the global context for decreasing the number of infected cases and deaths would require about “80 doses of vaccines per 100 inhabitants in order to sustain a decrease in confirmed cases and the number of deaths”. As was shown, approximately 47 doses of vaccines have to be administered in order to reduce infected cases when an intensive vaccination campaign was introduced at the beginning of the pandemic wave. However, the findings also indicate a need to increase the number of doses when the pandemic grew—data retrieved from May 2021 enabled them to conclude that an increase of COVID-19 wave required a higher optimal level of vaccines to be administered—it was estimated to be about 90 doses.

Finally, in the literature, there are also many interesting studies related to analyses of the impact of vaccinations and their combination with other non-pharmaceutical measures were presented. Among others, Viana et al. [17] studied the case of Portugal, while Maghadas et al. [18] and Coccia [19] provided evidence for the US.

The short literature review, presented above, strongly emphasises the fact that the assumptions concerning the size of the proportion of the immune population, the coverage of vaccinations or the reproduction number all affect herd immunity.

In our new study, we would like to present the results of virus spread simulations in three scenarios with immunity. We will also refer to the scenarios described in [1], which did not include immunity. The new scenarios took into account the process of vaccinating the population over time and its impact on the course of the pandemic. In the first scenario, approximately 50% of the population was vaccinated. In the second one, approximately 80% of the population was immune. In the last scenario, we present the conditions under which herd immunity can be expected in a relatively small economy, and therefore, we offer evidence about why lockdowns are still an important tool in the fight against a pandemic.

## 3. Updated ABM Component for Studying the Dynamics of the COVID-19 Pandemic

We updated the agent-based model that was presented in [1] in order to introduce immunity into the system (we present the details of the ABM model in Section 3). Using this ABM component, we simulated the spread of the COVID-19 virus and analysed the impact of the COVID-19 pandemic on society’s overall labour productivity within three scenarios that took into account both vaccinations and natural immunity. Those scenarios will be described in Section 4. In this section, the results that were obtained in 2020 in connection with the introduction of vaccination will be validated. Then, we will estimate the economic impact of the COVID-19 pandemic using the dynamic stochastic general equilibrium (DSGE) model (see Section 5).

We describe the functioning of the model in the following six modules. The way in which the program works is analogous to the one described in [1], but this time, the program and the analyses included the vaccinations and immunity of individuals who had previously contracted COVID-19. We listed the most important elements of the program and we emphasised the changes that had been made recently.

In the first module, the initial conditions were defined. The variables and parameters that had to be specified in order to run the simulations are presented in Table 1, Table 2 and Table 3.

We estimated the values of the parameters and the transition probabilities that would be assumed in a specific scenario using the empirical data. We present the calibration for a given scenario in Table 4 and Table 5.

The health status of an agent, their age and location were randomly assigned at the beginning of the simulation (the number of infected agents were set in the initial conditions). In the second module, the characteristics of the agents were recorded in the matrices after each time step. The simulation was conducted for the values of the parameters that had previously been defined. Among the most important characteristics that were recorded after each time step (weekly) were: the health status of each individual in a society (an M×T matrix $H^{*}$), the productivity of each individual in a society (an M×T matrix $W^{*}$), the age of each individual in a society (an M×T matrix $A^{*}$) and the location of each individual on the map after each iteration (*x*- and *y*-coordinates) (an M×2T matrix $X^{*}$). The full dataset was also recorded in the matrix (an M×4 matrix $F^{*}$).

Note that this approach is analogous to the one that we adopted in our study in November 2020. However, because we added two variables (recovered and vaccinated agents), this also affected the way the transition probabilities had to be defined. Therefore, the matrices $H^{*},W^{*},A^{*}$ and $X^{*}$ are different from the H,W,A,X that were presented in November 2020. Moreover, because the simulations were stochastic, the results that were recorded for each simulation also differed. In the article, we present the results that were averaged for 100 simulations.

The movements of the agents were described in the third module. The grid represented a closed economy. Although this simplification is easily modifiable and there is the possibility to introduce new infections from outside the economy, the aim of this study was to show the validity of lockdowns in the simplest way. The research results would be similar, even if this assumption was lifted. The logic that is known from the cellular automata models was adopted in the study, see [20,21]. We tested several neighbourhoods of a cell in which a healthy agent could move. As the adoption of a specific neighbourhood did not significantly affect the results, we present the conclusions for a simulation in which the agents can move around in the Moore neighbourhood of a cell, which was defined as a two-dimensional square lattice and was composed of a central cell and the eight cells that surround it. An infected agent (symptomatically or asymptomatically), while moving on the grid, encounters other agents and thus spreads the virus. Agents that are receiving treatment, in quarantine or are deceased stop moving on the grid. The size of the grid was carefully selected in order to represent the actual scaled empirical population density of the selected country.

In the fourth module, we defined how the virus can be spread in the society. The program analyses the neighbourhood of each individual and determines whether there is someone who might infect other agents.

*Cases for Healthy Individuals*

**Figure 1:**
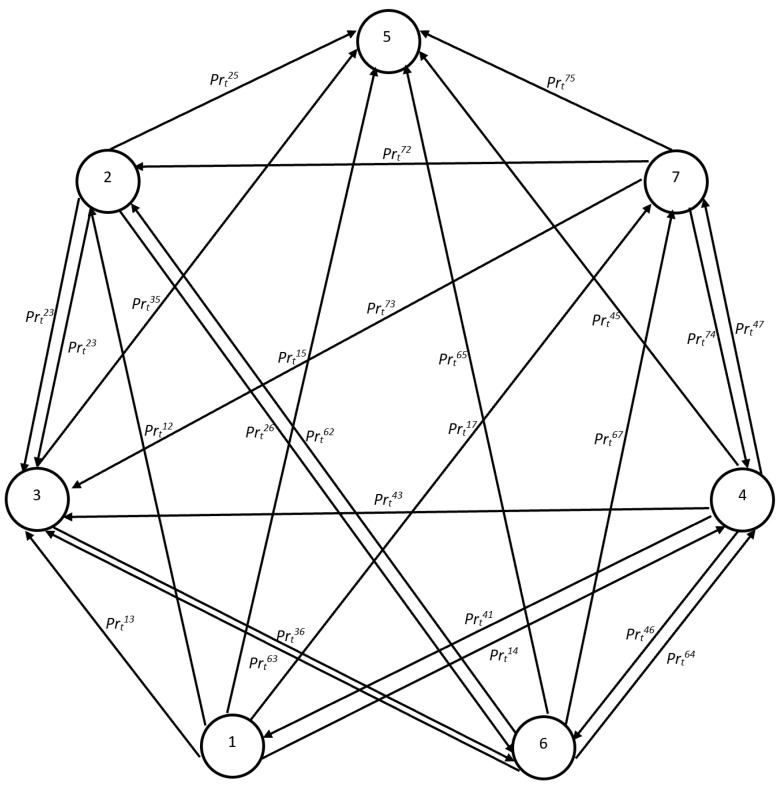
State transition probabilities in the agent-based epidemic component. Health status: 1—healthy (*h*), 2—infected (*i*), 3—treated (*t*), 4—healthy individuals in preventive quarantine (*q*), 5—deceased (*d*), 6—recovered (*r*), 7—vaccinated (*v*) $P^{ij}$—transition probability between states *i* and *j*, see Table 2 and Table 3.

The program determines whether there were any infected ($s_{t}^{Ind}=2$) or treated individuals ($s_{t}^{Ind}=3$) in the neighbourhood of a healthy agent ($s_{t}^{Ind}=1$). If there were, they could have been infected ($s_{t}^{Ind}=2$) with a certain probability. With a given probability, they could also have been treated in hospital (or put in isolation) ($s_{t}^{Ind}=3$). Infection was not equivalent to a diagnosis of sickness. This part of the program is based on two probabilistic tests. The first probabilistic test determined whether an individual had been infected. However, only the second one determined whether the individual had been diagnosed and had been receiving treatment. If an individual was not infected, they could still be directed into preventive quarantine ($s_{t}^{Ind}=4$) with a certain probability. There are also non-negative chances that a healthy agent might die within one week ($s_{t}^{Ind}=5$). If the system determined that no prior changes in status could be applied, then with a certain probability, a person could be vaccinated and remain healthy ($s_{t}^{Ind}=7$). The state transition probabilities in the agent-based epidemic component that included immunity are presented in Figure 1.

*Cases for Infected Individuals*

For all of the agents that were already infected ($s_{t}^{Ind}=2$), the program performed probabilistic tests that determined whether an agent should be referred for treatment ($s_{t}^{Ind}=3$) or whether they had managed to overcome the virus ($s_{t}^{Ind}=6$) (’recoveries’) or had died ($s_{t}^{Ind}=5$).

*Cases for Treated or Infected Individuals in Isolation*

With certain probabilities, an agent that was also receiving treatment ($s_{t}^{Ind}=3$) could change their state to recovered ($s_{t}^{Ind}=6$) or deceased ($s_{t}^{Ind}=5$). They could also remain in hospital or in isolation ($s_{t}^{Ind}=3$).

*Cases for Healthy Individuals in Preventive Quarantine*

For the agents in preventive quarantine ($s_{t}^{Ind}=4$), the program determined the length of time that an individual had remained in quarantine. There were two alternatives based on a probabilistic test. The individual could be released after two time steps (weeks) or an agent would have to remain in quarantine. If an agent was healthy after quarantine, the program would assign his prior state (healthy, recovered, vaccinated) with a certain probability: ($s_{t}^{Ind}=1$, $s_{t}^{Ind}=6$ or $s_{t}^{Ind}=7$). Moreover, a quarantined agent could have contracted the virus as a result of contacts during or at the end of the quarantine (respectively, states $s_{t}^{Ind}=3$ and $s_{t}^{Ind}=2$) with a certain probability. In the worst-case scenario, an agent could have died in isolation with a very low probability ($s_{t}^{Ind}=5$).

*Cases for Recovered Individuals*

These agents were treated in the same way as healthy ones. However, in their case, we assumed a decreased probability of them becoming infected or being hospitalised. In addition, recovered agents would become immune to COVID-19 for several weeks in all three scenarios.

*Cases for Vaccinated Individuals*

These agents were treated in the same way as recovered ones. However, we assumed a lower probability of them becoming infected or being hospitalised. In addition, vaccinated agents would become immune for a longer period of time than recovered patients.

In our stylised simulation, we attempted to take into account the most important characteristics that could affect the dynamics of the spread of the virus and the impact of the pandemic on an economy. Therefore, all of the probability tests considered the age of an individual. This is important because, according to the empirical data, in the first waves of the coronavirus, the elderly were more likely to suffer with a severe disease or die from the coronavirus. Apart from changes in the health status, one of the most important characteristics of the agents was productivity. When their health status changed, an agent’s productivity was updated accordingly. Any decrease in an agent’s productivity was extensively consulted with both medical specialists and economists. The calibration was also consistent with the conclusions that had been extracted from the literature.

In the fifth module, the aggregation for each iteration is performed. As a result, we obtained the overall number of:

- Healthy individuals by age for each iteration;
- Infected agents by age for each iteration;
- Recovered agents by age for each iteration;
- Vaccinated agents by age for each iteration;
- Individuals receiving treatment by age for each iteration;
- Agents in preventive quarantine by age for each iteration;
- Agents deceased by age for each iteration.

These data were used as the input data for the dynamic stochastic general equilibrium model, which will be described in the following sections.

The last part of the code helps us visualise the results of the simulations. It also permits the results to be systematised in csv tables for further analysis using the DSGE model. The most important input data were the productivity shock.

## 4. Validation of Scenarios in Connection with the Introduction of Vaccination

In Kaszowska-Mojsa and Włodarczyk (2020) [1], four scenarios were analysed. However, because it was the early stage of the COVID-19 pandemic, we did not assume immunity or the effects of vaccinations in our study.

In the first scenario in [1], we studied the spread of the coronavirus in a country that was under mild restrictions. Home isolation was compulsory in this scenario. We also assumed that in more severe cases, people would be hospitalised. In both cases, the agents spent at least three weeks there. However, agents who had contact with an infected individual were quarantined only with a given probability. The quarantine period was a minimum of two weeks. At the same time, no additional restrictions were assumed by the regulator. In 2020, we treated this scenario as a baseline scenario (1). In 2021, we updated this scenario in order to introduce different levels of population immunity into the model (see: scenarios 1.1., 1.2. and 1.3) and hence validated the results. We managed to prove that the conclusions that had been presented in [1] were still valid when the vaccination process and natural immunity after recovery were taken into account.

The results for scenarios 1.1, 1.2 and 1.3 were presented in relation to scenarios 1–4 from the research conducted in 2020 [1]. For ease of reading, we briefly described below the assumptions of scenarios 2–4 and the main conclusions. For specific calibration of scenarios 1–4, see Table 4. For more information, see [1].

**Table 4:** Comparison of the calibration of scenarios 1–4.

Notation	Scenario 1	Scenario 2	Scenario 3	Scenario 4
*T*	104	104	104	104
$N^{Ind}$	10,000	10,000	10,000	10,000
$K^{Ind}$	150	150	150	150
$S_{t}\times S_{t}$	100×100 for all *t*	Dynamic adjustment *	Dynamic adjustment *	100×100 for all *t*
${\left(Ag\right)}_{t}^{1}$	0.181	0.181	0.181	0.181
${\left(Ag\right)}_{t}^{2}$	0.219	0.219	0.219	0.219
${\left(Ag\right)}_{t}^{3}$	0.6	0.6	0.6	0.6
${\left(Wp\right)}_{t}^{av\_h}$	1 for all *t*	Dynamic adjustment *	Dynamic adjustment *	1 for all *t*
${\left(Wp\right)}_{t}^{av\_inf}$	0.9	0.9	0.9	0.9
${\left(Wp\right)}_{t}^{av\_q}$	0.8	0.8	0.8	–
${\left(Wp\right)}_{t}^{av\_t}$	0.3	0.3	0.3	0.3
${\left(Pr\right)}_{t}^{12}$	0.03	0.03	Dynamic adjustment *	0.2
${\left(Pr\right)}_{t}^{13}$	0.1	0.1	Dynamic adjustment *	0
${\left(Pr\right)}_{t}^{15}$	0.00002	0.00002	Dynamic adjustment *	0.00002
${\left(Pr\right)}_{t}^{21}$	0.6998	0.6998	Dynamic adjustment *	0.6998
${\left(Pr\right)}_{t}^{24}$	0.2	0.2	Dynamic adjustment *	0.2
${\left(Pr\right)}_{t}^{25}$	0.0002	0.0002	Dynamic adjustment *	0.005
${\left(Pr\right)}_{t}^{41}$	0.6	0.6	Dynamic adjustment *	–
${\left(Pr\right)}_{t}^{43}$	0.1	0.1	Dynamic adjustment *	–
${\left(Pr\right)}_{t}^{45}$	0.0002	0.0002	Dynamic adjustment *	–
${\left(Pr\right)}_{t}^{31}$	0.7	0.7	Dynamic adjustment *	0.7
${\left(Pr\right)}_{t}^{35}$	0.0002	0.0002	Dynamic adjustment *	0.002

* The details of dynamic adjustment were described in [1].

In the second scenario, we simulated the spread of the COVID-19 pandemic under mobility restrictions, i.e., we focused on the impact of a lockdown on the spread of the SARS-CoV-2 virus and on the economy. We assumed that the duration of a lockdown would be at least two months (the lockdown was relatively long). The main observation was that “an extreme lockdown resulted in the long-term decrease in productivity in the economy” [1]. However, the pre-crisis level of productivity was achieved within two years after the outbreak of the COVID-19 pandemic. There was no permanent loss of productivity due to “an increase in the number of deaths and the permanent destruction of jobs”.

In the third scenario, we studied the effects of gradually introducing preventive restrictions on a society, such as mobility restrictions, restrictions that could affect the probability of infection and a lockdown. Then, we analysed the impact of these restrictions on the spread of the virus and the economy.

In the fourth scenario, we described the situation in which the coronavirus spread in a society in a much more aggressive manner. At the same time, the death rate was also higher. In this scenario, no restrictions were imposed on a society by the government and the spread of the virus was unrestricted. No large-scale testing was performed. Quarantine or home isolation was not mandatory.

For each of the four scenarios, we generated the labour productivity paths using the agent-based epidemic component. We then used these to obtain conditional forecasts of the main macroeconomic indicators, i.e., output, capital and investments as well as the unemployment rate, see Section 5 and Section 6 (using the DSGE model).

**Figure 2:**
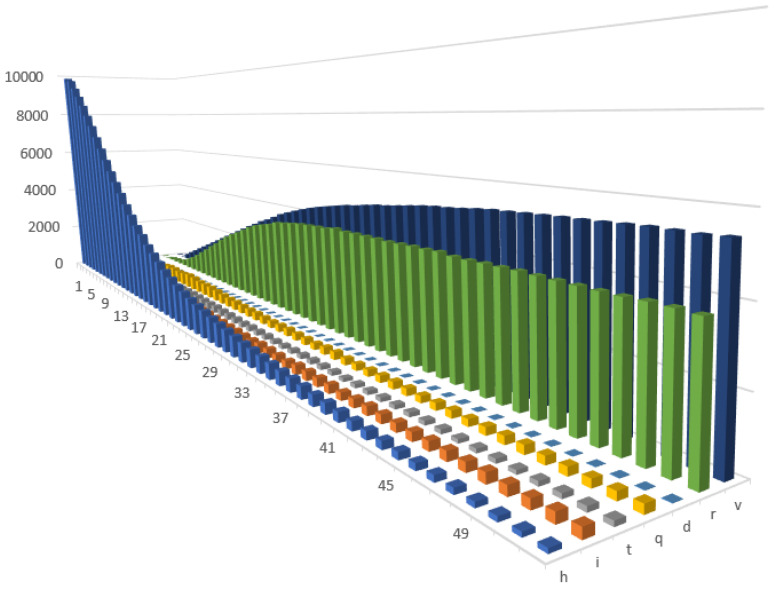
Changes in the health states in Scenario 1.1 (with immunity).

To validate the results after introduction of COVID-19 vaccine, we developed three new scenarios. In the first one (1.1), we updated the baseline scenario (1) with the immunity periods for vaccinated and recovered agents. Like in baseline scenario (1), we assumed the existence of only mild restrictions. In this scenario, the agents were vaccinated under certain probability (representing their willingness to get vaccinated) or under the condition that they were healthy or had recovered. In addition, there was a restriction on how many agents could be vaccinated in one period of time. We assumed that vaccinated agents would be immune for 20 weeks and that they would have a lower probability of being infected afterwards. Similarly, recovered people would be protected for ten weeks and would have lower a probability of being reinfected. Furthermore, our code enabled the period of immunity and transition probabilities to be modified. In this scenario, on average, we achieved up to 50% of immune agents in the population. In addition, we assumed that the effects of the vaccine would decrease over time. However, when the positive effects of vaccinations would be fading away, new individuals would be getting vaccinated. We also included the possibility of receiving additional dose of vaccine. For those reasons, we observed fluctuations in the number of infected people (as well as of the other states, see Figure 2) that translated into fluctuations in productivity.

**Figure 3:**
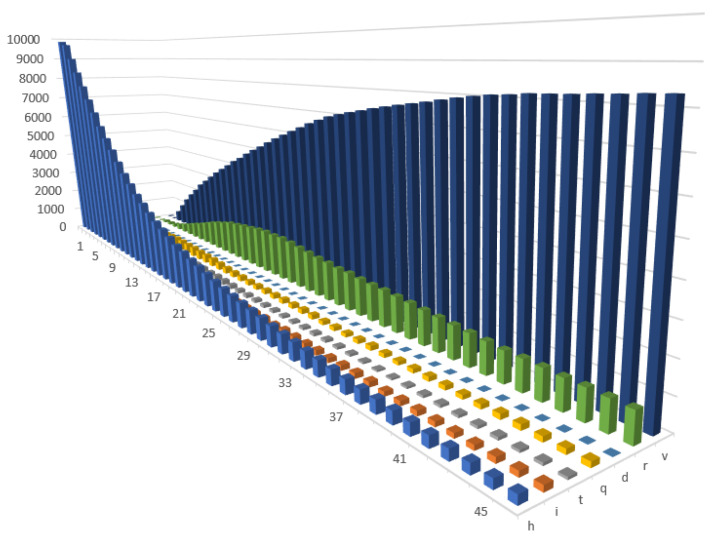
Changes in the health states in Scenario 1.2 (with immunity).

In the second scenario (1.2), we tested whether a higher percentage of immune agents in a society (80%) would enable herd immunity to be achieved and thus could avoid introducing further prevention and control measures. In order to obtain this higher percentage of vaccinated agents, we assumed that the vaccination process would be more effective (i.e., a larger number of agents could be vaccinated each day and we also increased probability of getting vaccinated. This probability of getting vaccinated would be higher, e.g., if there had been efficiently conducted pro-vaccination campaigns that increased public willingness to get vaccinated). In Figure 3, a gradual decrease in the number of agents in quarantine can be observed, however, a relatively high percentage of individuals were still being infected and hospitalised. Unfortunately, a large percentage of people also died. After recovery, immunity was gradually built up in a society. Building herd immunity is supported by the vaccination process, although it should be emphasised that herd immunity was not achieved. A higher vaccination coverage led to a lower number of infected individuals and a correspondingly fewer number of recoveries over time. Vaccinating agents at a higher level also reduced the burden on the health care system. Changes between the states would also update the states of the agents in terms of their productivity. Taken together, in the aggregate, we observed changes in the productivity shock, which then fed the DSGE model.

**Figure 4:**
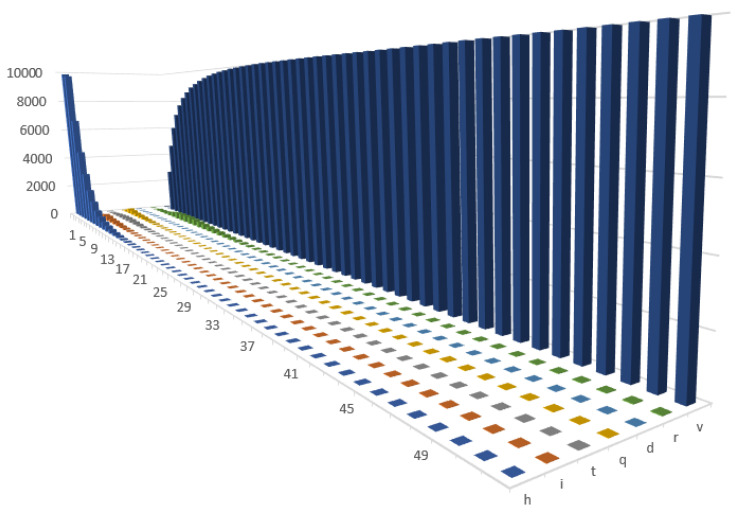
Changes in the health states in Scenario 1.3 (with immunity).

In the third scenario (1.3), we further increased the effectiveness of the vaccination process as well as the probability of getting vaccinated. We did this until we achieved herd immunity. This was achieved when approximately 90% of the population was vaccinated or had already recovered, see Figure 4. In all three scenarios, we observed a moderate decrease in productivity during the second year. This can be easily explained by the existence of a group of individuals who were against vaccinations and the other new forms of COVID-19 treatment. This trend also reflected the fact that part of population had died as a result of being infected with COVID-19 or from natural causes (for now, we did not allow new agents to be created in the model). In Table 5, the calibration for all three scenarios with vaccinations and immunity after recovery is compared.

**Figure 5:**
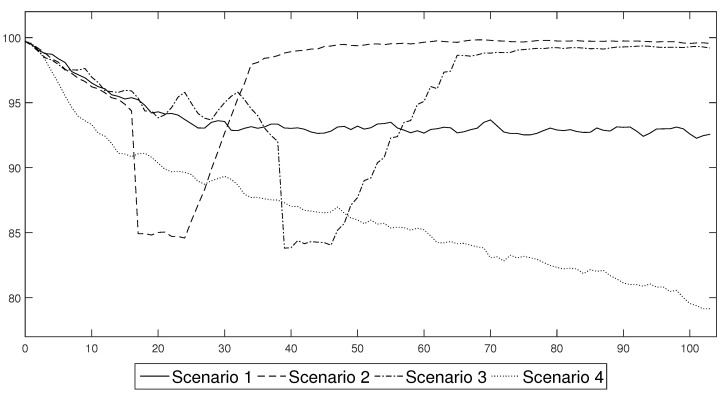
Aggregate labour productivity under the different COVID-19 prevention and control schemes. Please note that this figure is similar to the one that was published in [1] in November 2020. This figure enables the results for the scenarios that were analysed in 2021 to be compared with those from 2020.

In Figure 5, the labour productivity paths for four scenarios without immunity are presented.

**Figure 6:**
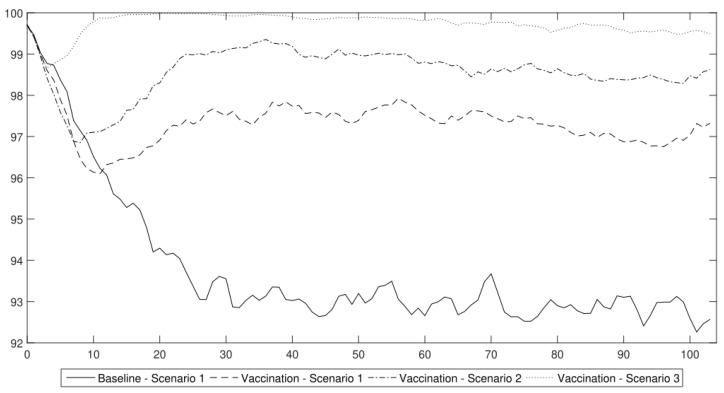
Aggregate labour productivity under the different COVID-19 vaccination schemes. Vaccination Scenario 1 is (1.1); Vaccination Scenario 2 is (1.2) and Vaccination Scenario 3 is (1.3).

In Figure 6, the productivity paths for all three scenarios with immunity are presented. It is easy to observe that all of the scenarios that included the vaccination process achieved much better results than the baseline scenario that had only standard preventive measures (face masks and quarantine). However, because approximately 90% of population needs to be vaccinated or has to recover in order to obtain herd immunity, the use of lockdowns seems to be indispensable. In the following sections, we used those productivity shocks as an input into the DSGE model in order to obtain a conditional forecast of the main macroeconomic variables, which would indicate the impact of vaccinations on the economy (i.e., output, investment, capital and unemployment rate).

**Table 5:** Comparison of the calibration of scenarios 1.1–1.3 (with immunity).

Notation	Scenario 1.1	Scenario 1.2	Scenario 1.3
*T*	104	104	104
$N^{Ind}$	10,000	10,000	10,000
$K^{Ind}$	150	150	150
$S_{t}\times S_{t}$	100×100 for all *t*	100×100 for all *t*	100×100 for all *t*
${\left(Ag\right)}_{t}^{1}$	0.181	0.181	0.181
${\left(Ag\right)}_{t}^{2}$	0.219	0.219	0.219
${\left(Ag\right)}_{t}^{3}$	0.6	0.6	0.6
${\left(Wp\right)}_{t}^{av\_h}$	1 for all *t*	1 for all *t*	1 for all *t*
${\left(Wp\right)}_{t}^{av\_inf}$	0.9	0.9	0.9
${\left(Wp\right)}_{t}^{av\_q}$	0.8	0.8	0.8
${\left(Wp\right)}_{t}^{av\_t}$	0.3	0.3	0.3
${\left(Pr\right)}_{t}^{12}$	0.03	0.03	0.03
${\left(Pr\right)}_{t}^{13}$	0.1	0.1	0.1
${\left(Pr\right)}_{t}^{14}$	0.1	0.1	0.1
${\left(Pr\right)}_{t}^{15}$	0.00002	0.00002	0.00002
${\left(Pr\right)}_{t}^{17}$	0.05	0.05	0.3
${\left(Pr\right)}_{t}^{23}$	0.2	0.2	0.2
${\left(Pr\right)}_{t}^{25}$	0.0002	0.0002	0.0002
${\left(Pr\right)}_{t}^{26}$	0.6998	0.6998	0.6998
${\left(Pr\right)}_{t}^{35}$	0.0002	0.0002	0.0002
${\left(Pr\right)}_{t}^{36}$	0.7	0.7	0.7
${\left(Pr\right)}_{t}^{41}$	0.6	0.6	0.6
${\left(Pr\right)}_{t}^{43}$	0.1	0.1	0.1
${\left(Pr\right)}_{t}^{45}$	0.0002	0.0002	0.0002
${\left(Pr\right)}_{t}^{46}$	0.06	0.06	0.06
${\left(Pr\right)}_{t}^{47}$	0.06	0.06	0.06
${\left(Pr\right)}_{t}^{62}$	0.01	0.01	0.01
${\left(Pr\right)}_{t}^{63}$	0.0005	0.005	0.005
${\left(Pr\right)}_{t}^{64}$	0.05	0.05	0.05
${\left(Pr\right)}_{t}^{65}$	0.00001	0.00001	0.00001
${\left(Pr\right)}_{t}^{67}$	0.009	0.1	0.2
${\left(Pr\right)}_{t}^{72}$	0.009	0.005	0.005
${\left(Pr\right)}_{t}^{73}$	0.00045	0.00025	0.00025
${\left(Pr\right)}_{t}^{74}$	0.05	0.05	0.05
${\left(Pr\right)}_{t}^{75}$	0.00001	0.00001	0.00001

## 5. Macroeconomic Consequences of Pandemics—The DSGE Approach

Like in [1], we also used the DSGE model to assess the macroeconomic consequences of the COVID-19 pandemic under the different prevention and control schemes, with a special emphasis on vaccinations. We used the approach that enabled the business cycles of modern economies to be replicated. The model was based on the model that was elaborated by Gali [22]. However, our aim was to extend it in a such way that the capital accumulation and labor market components could be introduced. We developed our model in line with the works of Christiano et al. [23], Gali [24,25], Gali et al. [26]. A description of the equations that were used in the modelling process can be found in our previous paper [1]. The changes in the calibration are explained in this section.

In order to study the impact of the COVID-19 pandemic on the economy using this framework, we introduced an additional labour productivity shock into the DSGE model. This shock was obtained from the agent-based component that was previously described. This approach enabled us to assess the consequences of a change in the availability of employees because they were infected, hospitalized, quarantined and because of the introduction of remote work. A change in their health status or working remotely made employees less effective or prevented them from working at all. It should be noted that these employees continued to work for the company in question and received either wages or sickness benefits for this work. Therefore, the COVID-19 shock should not be considered to be a labour supply shock, which pushes part of the labour force into inactivity as was the case in [27]. In our view, illness may have caused employees to be unproductive or not fully productive, but in many cases, it did not have any negative impact on the formal employment relationship. Such an approach located the first impact of a COVID-19 pandemic on the supply side of an analysed economy, which led to better reproducing the character of the pandemic disturbances. The demand-side effects were a second-order phenomenon. Such an approach is also in line with the results of the research on the nature of pandemic shock that assesses the supply-side effects as being the major factor that is responsible for the economic disturbances that have been caused by the SARS-CoV-2 pandemic [28].

The model assumed that “an economy was populated by a unit mass continuum of households that maximised their utility levels by solving the optimisation problem” as was described in [1]. The model is expressed in weekly terms in order to be able to study the dynamics of the COVID-19 pandemic. This approach was also used in [27,29]. In Table 6, the calibration of the model is presented. It was performed in such a way so that it matches the standard stylised facts associated with the business cycles of developed economies. Our model successfully reproduced the results of the empirical studies such as, for example, the estimated model of Christiano et al. [30].

The model was slightly recalibrated with respect to [1] based on the fact that new information about the COVID-19 pandemic was provided. We assumed a discount factor β=0.9996, which resulted in a steady-state interest rate of 2.1% in annual terms. Following the approach adopted by Christiano et al. [30] and Gali [25], we set the expected duration of prices and wages to 52 weeks, which makes $\theta_{p}=\theta_{w}=0.9807$. As in the study of in Gali [25], we assumed that $\epsilon_{w}=4.52$ and φ=5. As a result of the adopted calibration, the steady-state unemployment rate was approximately 4.8%. In our model, the unemployment rate could be identified with the natural unemployment rate under certain restrictions. The habit persistence parameter, *h*, was set at a relatively high level of 0.9. Nonetheless, this value is acceptable if we consider the fact that we adopted the calibration in weekly terms. As was expected, consumption was characterised by a relatively high week-to-week inertia. We calibrated the capital share in production to 0.25 (α=0.25). Following the analysis of the empirical data concerning the behaviour of capital during the pandemic, we decided to slightly recalibrate the parameters concerning the capital accumulation $\phi_{k}=12$ and δ=0.0175 (compared to $\phi_{k}=8$ and δ=0.05 in [1]). Both parameters enabled us to obtain the reactions of capital and investment that were better fitted to the actual tendencies that were observed in the data. These values also permitted the model to be identified.

Because the model was calibrated in weekly terms, the parameters of the Taylor rule also had to be adjusted. We assumed $\phi_{\pi }=0.115$ and $\phi_{y}=0.0096$. This calibration is consistent with the values of 1.5 and 0.125 in quarterly terms, respectively. Finally, we had to recalibrate the autoregressive parameters of the shocks in order to obtain the duration of the shocks in weekly terms. As a result, the values of $\rho_{a}=\rho_{\chi }=\rho_{N}=0.99$ and $\rho_{M}=0.965$ were assumed. The main advantage of the proposed calibration is that the Blanchard–Kahn conditions were fulfilled and the model could be identified. In both this article and in the research that was conducted in November 2020, the model was solved in nonlinear terms (no log-linearisation around the steady state was required).

## 6. COVID-19 Prevention and Control Schemes—What Does the Vaccination Change?

**Figure 7:**
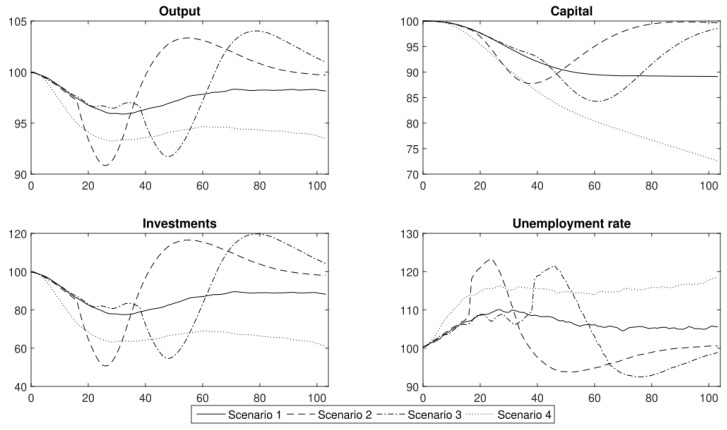
The major macroeconomic indicators under the different COVID-19 prevention and control schemes (conditional forecasts using the DSGE model). Please note that this figure is similar to the one that was published in [1] in November 2020. However, the capital accumulation process was recalibrated in the DSGE model as is explained in Section 5. This figure enables the results for scenarios analysed in 2021 to be compared with those from 2020.

Using the DSGE model and the labour productivity shocks that had been obtained from the agent-based epidemic component, we generated conditional forecasts of the standard macroeconomic indicators: output, capital, investments and the unemployment rate. In Figure 7, we present the results of the four scenarios without immunity (for a description of the scenarios, see Section 4). The analyses were performed for 104 weeks (two years). The results are expressed as the relative difference from the steady-state value. A mean of 10,000 simulations of the model is reported.

Our analysis in November 2020 showed that the scenarios could easily be divided into two groups, that produced similar economic trends. The first group consisted of scenarios 1 and 4, which “resulted in the occurrence of negative economic trends that persisted in an economy in the medium or even long term”. The other group was composed of scenarios with lockdowns (2 and 3). The use of lockdowns led to a deeper response of macroeconomic variables, but the negative effects were observable over a shorter period of time.

The first group consisted of “the scenarios that assumed that the government permitted the persistent spread of the disease by introducing only general sanitary restrictions (scenario 1) or by not introducing any restrictions at all and hoping that the propagation of the virus would finally cease at some point (scenario 4). Both of these approaches resulted in a relatively high share of people who were either infected or were placed in quarantine, which translated into a persistent decrease in the productivity of labour” [1].

In the case of the first scenario, the labour productivity stabilised at a level of approximately 92% of full capacity. In this scenario, we observed that the output initially decreased to approximately 97.5% of the steady value. Nonetheless, then it stabilised at 98% of its steady-state value. At the same time, it should be emphasised that there was a decrease in capital and investment by approximately 10% during the first year after the outbreak of the COVID-19 pandemic. During the second year after the onset of the pandemic, the unemployment rate stabilised at 5 pp. above the steady state, which translated into an actual unemployment rate of approximately 9%.

After the introduction of vaccinations, we modified this scenario to include immunity. We will present the results after commenting on the main results that were obtained from the scenarios 2–4 in 2020 in order to facilitate the reference of the results to the original study that we wanted to validate.

The strategy that enabled the virus to spread without restriction led to a permanent decrease in productivity to a level of 80% within two years after the outbreak of the COVID-19 pandemic that was described in scenario 4. In this scenario, output decreased by approximately 4% in the first half of the year. Then, it stabilised for another six months. During the second year, the output continued in a downward trend and reached approximately 94% of the steady-state value. While the output decreased, firms stopped making further investments. As a result, the level of investment was lower and the capital decreased as well. The unemployment rate increased by approximately 15 pp. within the next two years. We estimated a vast social cost as the actual unemployment rate reached 20%. These estimations did not include the long-term effects, i.e., due to the loss of human capital. The results of our analysis clearly show that the policy makers should not have followed the strategy of no reaction.

In scenarios 2 and 3, we compared the strategy of a strict lockdown with a gradual one. In both cases, the macroeconomic variables (output, capital, investment and unemployment rate) decreased by almost the same amount, see Figure 7. A lockdown that lasted for two months caused a contraction of output and economic activity that disappeared within six months. The main difference between scenarios 2 and 3 was the duration of the economic downturn that was caused by a lockdown. Gradual lockdowns unnecessarily prolonged the duration of restrictions as well as the adverse effects that were caused by the introduction of the prevention and control schemes, and thus seems to be suboptimal compared to an immediate action strategy.

To summarise, the main conclusion was that “if we decide to shape our policy according to scenarios 2 or 3, the changes in economic activity might be abrupt but short-lived. In the case of scenarios 1 or 4, the decrease in economic activity might not be as deep but would be rather permanent” [1]. However, the question of whether in the era of widespread vaccination, it would not make sense to follow the first baseline scenario instead of continuing to implement costly lockdowns remains? As we show, our results are still valid despite the introduction of a vaccine against COVID-19.

**Figure 8:**
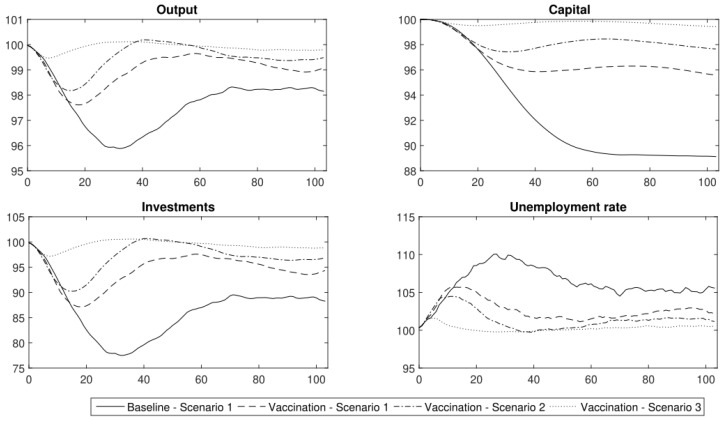
The major macroeconomic indicators under the different COVID-19 vaccination schemes (conditional forecasts using the DSGE model). Vaccination Scenario 1 is (1.1); Vaccination Scenario 2 is (1.2) and Vaccination Scenario 3 is (1.3).

In Figure 8, the results of three scenarios with immunity (1.1–1.3) compared to the baseline scenario (1) are presented. In the first scenario with immunity (1.1), the output initially dropped by at least 2%. For the following 1.5 year, the output stabilised at 98.5% of its steady-state value. In this scenario, the contraction of capital and investment (of approx. 4%) was permanent. In the first 25 weeks, the unemployment rate increased by 5 pp. Then, it stabilised at 2.5 pp. above the steady state.

In the second scenario with immunity (1.2), we observe that the output decreased by at least 1.5% in the first 20 weeks. In the next few weeks, it returned to a level of approximately 99% of its steady-state value. There was a permanent decrease in capital and investment of approximately 2%. The situation on the labour market deteriorated in this first period (the unemployment rate was greater by 3 pp. during that period). However, in the second half of the year of the COVID-19 pandemic, the unemployment rate stabilised at 2 pp. above the steady state.

Only in the third scenario with immunity (1.3) was the decrease in output temporary and negligible in the long term. Capital and investment decreased but the contraction was not permanent. The social costs were also low. The unemployment rate increased in the first weeks of the pandemic but decreased after a society had been vaccinated (after obtaining immunity at a level of 90%).

To conclude, the use of lockdowns is still an effective strategy that we should use because a vaccination coverage of 0.5 (50% of the population) does not contain the spread of the virus enough. Herd immunity was only achieved when the vaccination coverage was very high (approximately 90% of the society needs to be immune). Such a high vaccination coverage of the population is virtually impossible when people’s skepticism towards vaccinations is considered and the presence of contraindications to vaccinations (i.e., children under five years of age cannot be vaccinated and certain diseases are also contraindications).

## 7. Conclusions

Our analysis showed that vaccines and after-recovery immunity do change the dynamics of a contagion or reduce the adverse effects of pandemics on an economy. Although the consecutive pandemic waves have developed some self-limiting characteristics, we proved that it is hard to develop “herd immunity”. Even when vaccines are available, the disease remains a constant feature of the economic landscape, which causes losses in economic welfare. The outcomes that resemble “herd immunity” might only be generated for very high vaccination rates (i.e., approximately 90% of the population should be vaccinated or should obtain natural immunity).

The introduction of vaccines in the analysis did not change the main conclusions of the research that was conducted in November 2020. In the article, we showed that the changes in labour productivity that were caused by the spread of disease still lead to negative changes of the macroeconomic aggregates. Despite the fact that the magnitude of these changes is much smaller than in the case in which people did not vaccinate at all, the pandemic still depresses economic activity over a relatively long period of time. As the resulting economic fluctuations are not very abrupt, strategies that promote vaccination do permit the economic costs of pandemic to be reduced in terms of output losses, capital depreciation and unemployment. On the other hand, they prevent the occurrence of an economic recovery after the peak of pandemic wave as the labour productivity does not reach the steady state level.

A lockdown strategy causes bigger falls of productivity, output and capital, which are accompanied by increases in unemployment, in the initial phase, when harsh constraints on personal and economic activity are introduced. On the other hand, as the constraints are lifted, an economic recovery is observed. That recovery is a representation of the “creative destruction” phenomenon, which leads to the occurrence of microcycles of capital working towards an improvement of the economic perspectives after the lockdown. As a result, lockdowns do not extend the duration of a recession, which confirms that they are still a viable alternative in the fight against an epidemic.

## Figures and Tables

**Table 1:** The list of initial conditions to be set.

Initial Conditions	Explanation	Restrictions
*T*	Number of time steps (weeks)	≥0
$s_{t}^{Ind}$	Health status of the individual at time t=0 (1—healthy, 2—infected, 3—treated, 4—healthy individual in preventive quarantine, 5—deceased; 6—recovered, 7—vaccinated)	Int ∈{1,2,3,4,5,6,7}
${\left(Age\right)}_{t}^{Ind}$	Age of an individual at time t=0	
$N^{Ind}$	Number of individuals at time t=0	Int ≥0
$K^{Ind}$	Number of infected individuals at time t=0 (including asymptomatically infected)	Int ≥0
$\lambda^{max}$	The parameter corresponding to the maximum number of vaccinated persons in the iteration (week)	Int ≥0
$S_{t}\times S_{t}$	Dimensions of the grid at time *t* *	Int ≥0
${\left(Ag\right)}_{t}^{1}$	Share of citizens of pre-working age at time *t*	∈〈0,1〉
${\left(Ag\right)}_{t}^{2}$	Share of citizens of working age at time *t*	∈〈0,1〉
${\left(Ag\right)}_{t}^{3}$	Share of retired individuals at time *t*	∈〈0,1〉
${\left(Wp\right)}_{t}^{av\_h}$	The productivity of an individual when healthy at time *t* (it was assumed to be equal to one)	∈〈0,1〉
${\left(Wp\right)}_{t}^{av\_inf}$	The productivity of an individual when infected at time *t* (the decline in productivity was estimated based on empirical data)	∈〈0,1〉
${\left(Wp\right)}_{t}^{av\_r}$	The productivity of an individual after recovery at time *t* (the decline in productivity was estimated based on empirical data)	∈〈0,1〉
${\left(Wp\right)}_{t}^{av\_t}$	The productivity of an individual when treated or who is infected and in quarantine at time *t* (the decline in productivity was estimated based on empirical data)	∈〈0,1〉
${\left(Wp\right)}_{t}^{av\_q}$	The productivity of an individual who is healthy and in quarantine at time *t* (the decline in productivity was estimated based on empirical data)	∈〈0,1〉
${\left(Wp\right)}_{t}^{av\_v}$	The productivity of an individual who has been vaccinated at time *t* (it was assumed to be equal to one)	∈〈0,1〉

* The dimensions do not have to be constant in all scenarios for all *t*. We assumed that in baseline scenario and in scenarios with immunity *S_t_* = *S*.

**Table 2:** Probabilities that are set as parameters *.

Parameter	Explanation	Restrictions
${\left(Pr\right)}_{t}^{12}$	The probability that a healthy agent (1) will become infected (2) at time *t*	∈(0,1)
${\left(Pr\right)}_{t}^{14}$	The probability that a healthy agent (1) will be in quarantine (although she is healthy) (4) at time *t*	∈(0,1)
${\left(Pr\right)}_{t}^{15}$	The probability that a healthy agent (1) will become infected and will die almost instantly (within week) (5)	∈(0,1)
${\left(Pr\right)}_{t}^{17}$	The probability that the healthy agent (1) will be vaccinated (7)	∈(0,1)
${\left(Pr\right)}_{t}^{26}$	The probability that an infected agent (2) will become healthy (will recover) (6)	∈(0,1)
${\left(Pr\right)}_{t}^{23}$	The probability that an infected agent (2) will be treated in a hospital or will stay in quarantine (3)	∈(0,1)
${\left(Pr\right)}_{t}^{25}$	The probability that an infected agent (2) dies (5)	∈(0,1)
${\left(Pr\right)}_{t}^{35}$	The probability that an infected agent in a hospital or quarantine (3) dies (5)	∈(0,1)
${\left(Pr\right)}_{t}^{36}$	The probability that an infected agent in a hospital or quarantine (3) gets better (6) (recovers)	∈(0,1)
${\left(Pr\right)}_{t}^{41}$	The probability that a healthy agent in quarantine (4) will end the quarantine, that is, is healthy (1)	∈(0,1)
${\left(Pr\right)}_{t}^{43}$	The probability that a healthy agent in quarantine (4) will become infected during the quarantine and she is still in quarantine (but now is already infected) (3) at time *t*	∈(0,1)
${\left(Pr\right)}_{t}^{45}$	The probability that a healthy agent in quarantine (4) dies (5)	∈(0,1)
${\left(Pr\right)}_{t}^{46}$	The probability that a healthy agent in quarantine (4) was not infected and returned to the state “recovered” (6)	∈(0,1)
${\left(Pr\right)}_{t}^{47}$	The probability that a healthy agent in quarantine (4) was not infected and returned to the state “vaccinated” (7)	∈(0,1)
${\left(Pr\right)}_{t}^{61}$	The probability that the recovered agent (6) will get infected (1)	∈(0,1)
${\left(Pr\right)}_{t}^{64}$	The probability that the recovered agent (6) will go to the quarantine (4)	∈(0,1)
${\left(Pr\right)}_{t}^{65}$	The probability that the recovered agent (6) will die (5)	∈(0,1)
${\left(Pr\right)}_{t}^{67}$	The probability that the recovered agent (6) will get vaccinated (7)	∈(0,1)
${\left(Pr\right)}_{t}^{72}$	The probability that the vaccinated agent (7) will get infected (2)	∈(0,1)
${\left(Pr\right)}_{t}^{74}$	The probability that the vaccinated agent (7) will go to the quarantine (4)	∈(0,1)
${\left(Pr\right)}_{t}^{75}$	The probability that the vaccinated agent (7) will die (5)	∈(0,1)

* Estimated on empirical data.

**Table 3:** Variables and parameters that were computed by the program after each iteration.

Variable	Explanation	Restr.
${\left(Pr\right)}_{t}^{13}$	The probability that a healthy agent (1) will become treated in the hospital (or isolation) after becoming infected (3) at time *t*	∈(0,1)
${\left(Pr\right)}_{t}^{42}$	The probability that a healthy agent in quarantine (4) will become infected at the end of her quarantine at time *t*	∈(0,1)
${\left(Pr\right)}_{t}^{63}$	The probability that a recovered agent (6) will be hospitalised (3) at time *t*	∈(0,1)
${\left(Pr\right)}_{t}^{73}$	The probability that a vaccinated agent (7) will be hospitalised (3) at time *t*	∈(0,1)
*p*	Temporal variable that defines a threshold probability 1	∈(0,1)
*q*	Temporal variable that defines a threshold probability 2	∈(0,1)
*r*	Temporal variable that defines a threshold probability 3	∈(0,1)
*z*	New temporal variable that defines a threshold probability 4	∈(0,1)
$s_{t}^{Ind}$	Health status of the agent at time t>0 (1—healthy, 2—infected, 3—treated, 4—healthy individual in preventive quarantine, 5—deceased, 6—recovered, 7—vaccinated)	Int ∈{1,2,3,4,5,6,7}
${\left(Age\right)}_{t}^{Ind}$	Age of an agent at time t>0	≥0
${\left(Wp\right)}_{t}^{Ind}$	Productivity of an agent at time t>0	∈〈0,1〉

**Table 6:** Proposed calibration of the parameters of the model.

Variable	Description	Calibrated Values
A	Elasticity of output towards the changes of labour	0.25
φ	Reverse of the labour supply elasticity	5
$\epsilon_{w}$	Elasticity of substitution between types of labour	4.52
$\epsilon_{p}$	Elasticity of substitution between types of goods	9
$\theta_{w}$	Calvo index of wage rigidity	0.9807
$\theta_{p}$	Calvo index of price rigidity	0.9807
β	Discount factor	0.9996
δ	Capital depreciation rate	0.0175
$\phi_{k}$	Capital adjustment costs’ scaling parameter	12
*h*	Habit persistence parameter	0.9
$\rho_{a}$	Autoregressive parameter of the technological shock	0.99
$\rho_{\chi }$	Autoregressive parameter of the labour supply shock	0.99
$\rho_{a}$	Autoregressive parameter of the technological shock	0.99
$\rho_{\chi }$	Autoregressive parameter of the labour productivity shock	0.99
$\rho_{M}$	Autoregressive parameter of the monetary policy shock	0.965
$\phi_{\pi }$	Central bank’s reaction to the deviation of inflation from its steady state value	0.115
$\phi_{y}$	Central bank’s reaction to the deviation of output gap from its steady state value	0.0096

## Data Availability

Not applicable.

## Abbreviations

The following abbreviations are used in this manuscript:

ABM	Agent-Based Modelling
COVID-19	Coronavirus Disease 2019
DSGE	Dynamic Stochastic General Equilibrium
pp.	percentage points
SARS-CoV-2	Severe acute respiratory syndrome coronavirus 2
